# Supplementary material for: Neural representation of nouns and verbs in congenitally blind and sighted individuals
Source: Nat Commun. 2025 Aug 29;16:8090. doi: 10.1038/s41467-025-63423-0 (PMC12397302; doi:10.1038/s41467-025-63423-0)
Supplement: Supplementary file 1 — Supplementary Information [file 41467_2025_63423_MOESM1_ESM.pdf]

Supplementary Information

Neural representation of nouns and verbs in congenitally blind and sighted individuals

Marta Urbaniak, Małgorzata Paczyńska, Alfonso Caramazza, Łukasz Bola

Supplementary Figures

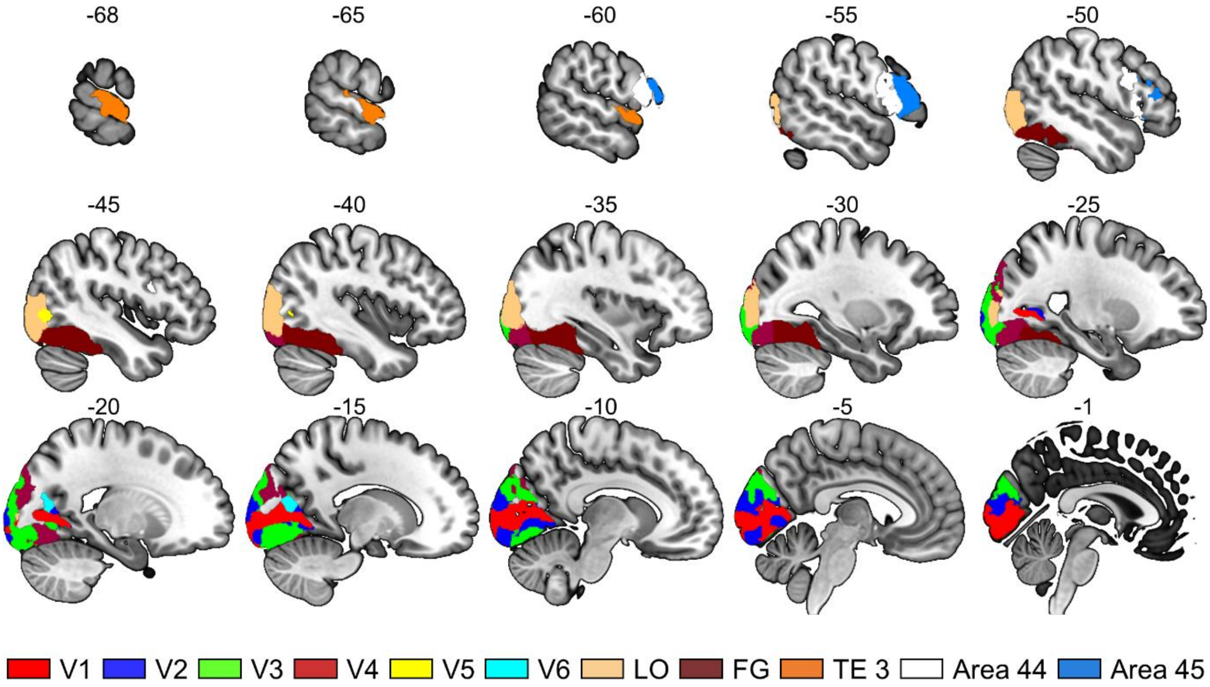

Supplementary Figure 1. Masks of brain areas used in the region-of-interest analysis. LO - the lateral occipital area; FG - the fusiform gyrus. The masks were visualized using MRIcroGL (https://www.nitrc.org/projects/mricrogl).

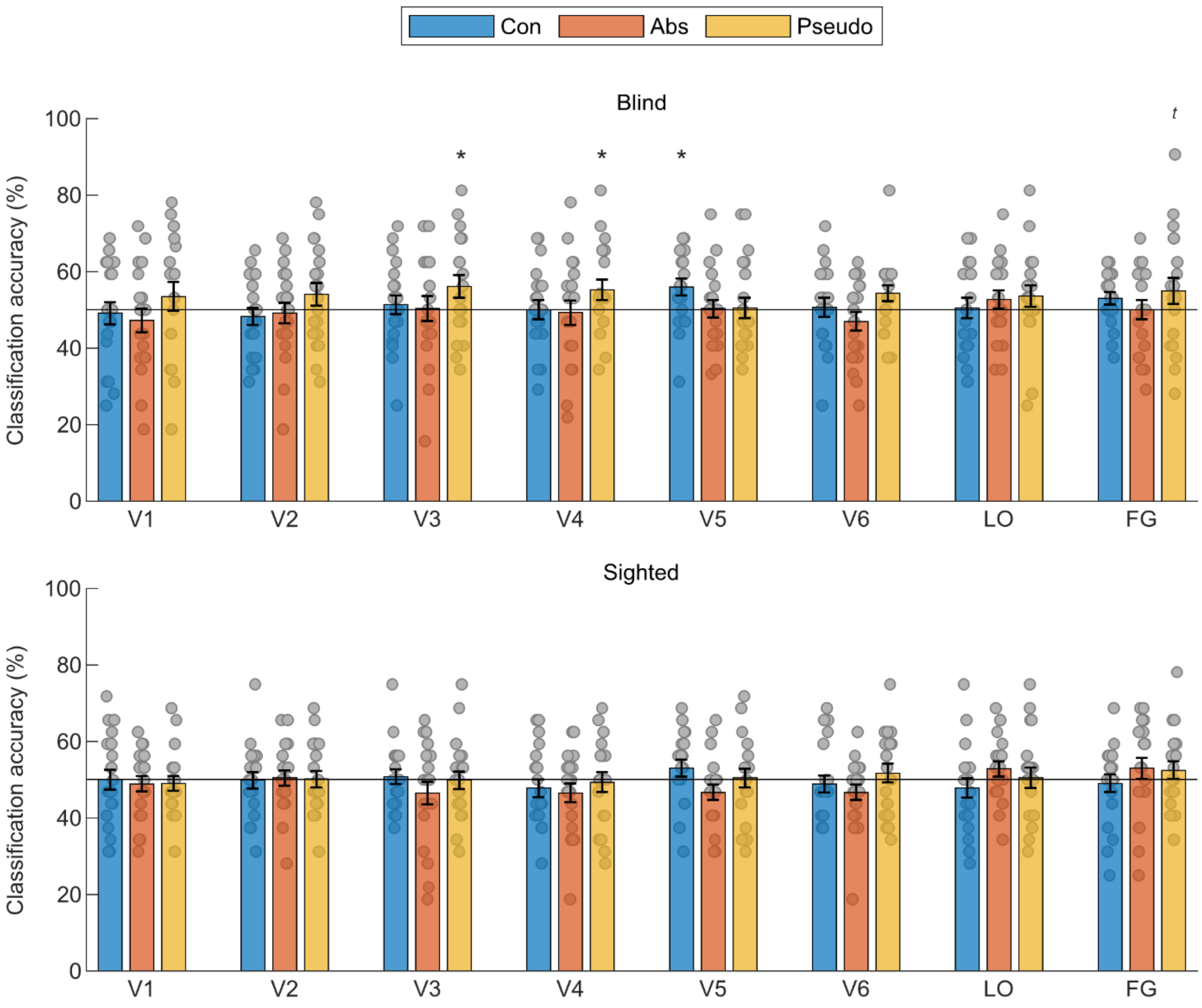

Supplementary Figure 2. Classification of activity patterns for nouns and verbs from specific semantic categories in all visual areas included in the study. Results of support vector machine classification of activity patterns for noun blocks and verb blocks performed separately for concrete, abstract, and pseudo word categories in the visual areas in congenitally blind (n = 20) and sighted (n = 20) participants. LO - the lateral occipital area; FG - the fusiform gyrus. * $p < 0.05$, $^{t}p = 0.051$. Statistical testing against classification chance level was performed separately in each participant group using the permutation procedure, in which the actual results were compared with the null distribution of 1000 classification values obtained with the labels of noun blocks and verb blocks randomly reassigned. The results were corrected for multiple comparisons across the word categories, within each group and visual area (Bonferroni correction for 3 tests). Error bars represent the standard error of the mean calculated across the results for individual participants in each group. The a priori chance classification level (accuracy = 50%) is marked with the black line. Source data and exact p-values are provided as a Source Data file.

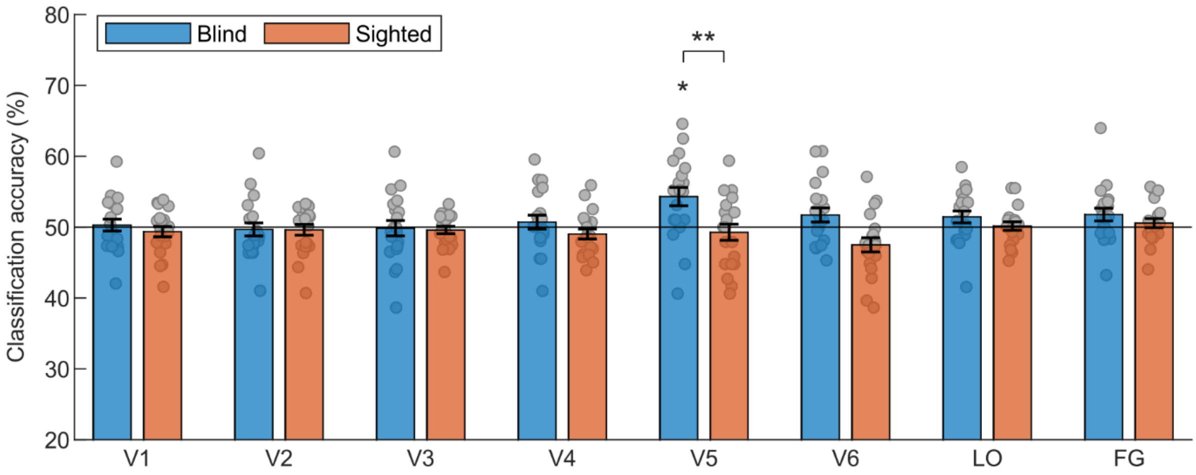

Supplementary Figure 3. Classification of activity patterns for nouns and verbs in the visual areas – analysis in masks with equated number of voxels. Results of support vector machine classification of activity patterns for noun blocks and verb blocks in the visual areas in congenitally blind (n = 20) and sighted (n = 20) participants. For each visual region, except for area V5/MT, the subsets of 193 voxels were randomly drawn from this region's mask. The classification was then performed in these subsets. There were 1000 iterations of this analysis, for each participant, and the results were averaged across the iterations. Since the mask of area V5/MT contained only 193 voxels, the results for the whole mask are presented. LO - the lateral occipital area; FG - the fusiform gyrus. * $p < 0.05$, ** $p < 0.01$. Statistical testing against classification chance level was performed separately in each participant group using one-tailed, one-sample t-test. The results were corrected for multiple comparisons across the visual areas, within each group (Bonferroni correction for 8 tests). The between-group test was performed only in area V5/MT, in which significant results were observed in the blind group, using the two-tailed, two-sample t-test. Since only one between-group comparison was performed, the correction for multiple comparisons was not necessary. Error bars represent the standard error of the mean calculated across the results for individual participants in each group. The chance classification level (accuracy = 50%) is marked with the black line. Source data and exact p-values are provided as a Source Data file.

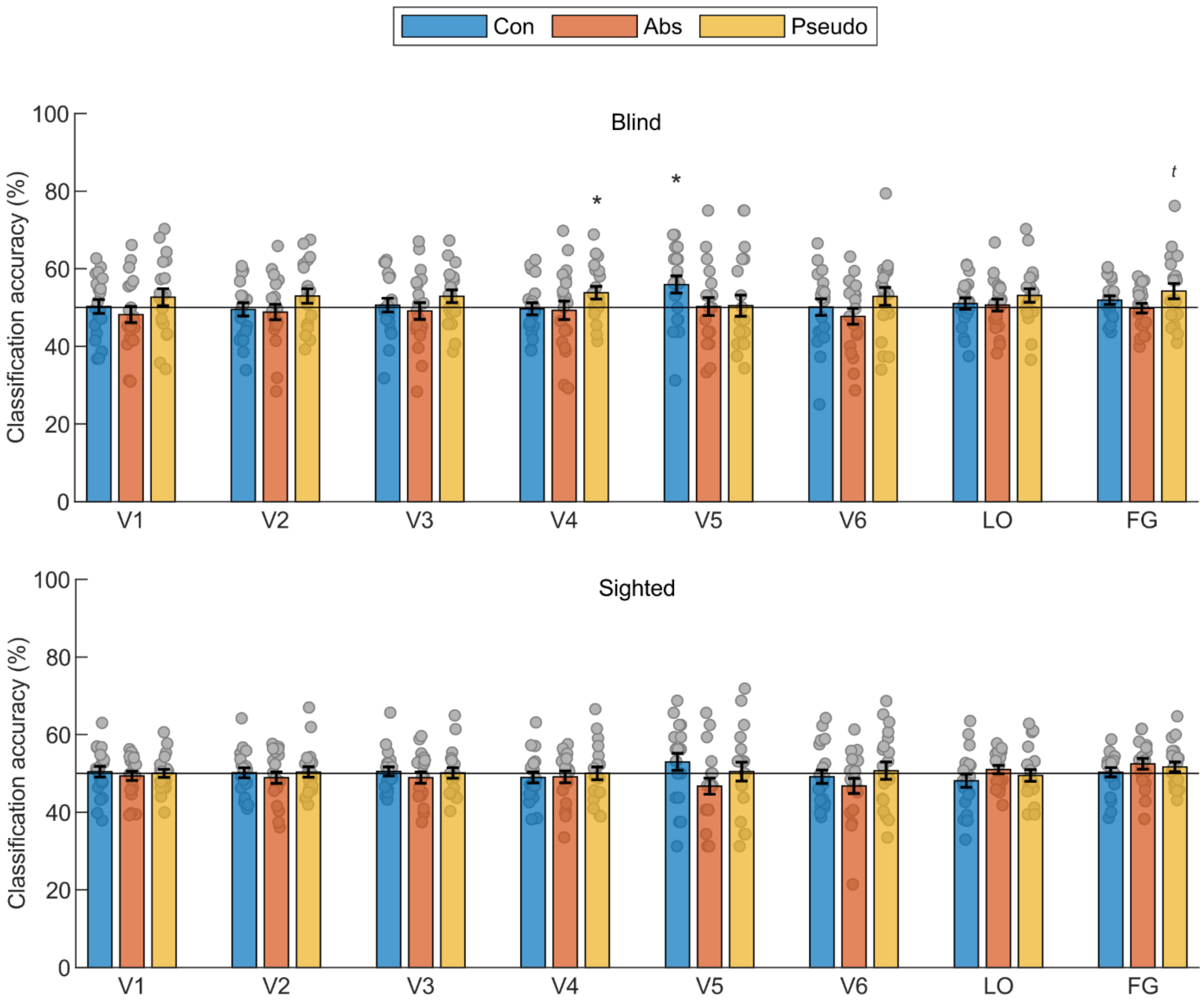

Supplementary Figure 4. Classification of activity patterns for nouns and verbs from specific semantic categories in the visual areas – analysis in masks with equated number of voxels. Results of support vector machine classification of activity patterns for noun blocks and verb blocks, performed separately for concrete, abstract, and pseudo word categories, in the visual areas in congenitally blind (n = 20) and sighted (n = 20) participants. For each visual region, except for area V5/MT, the subsets of 193 voxels were randomly drawn from this region's mask. The classification was then performed in these subsets. There were 1000 iterations of this analysis, for each participant, and the results were averaged across the iterations. Since the mask of area V5/MT contained only 193 voxels, the results for the whole mask are presented. LO - the lateral occipital area; FG - the fusiform gyrus. * $p < 0.05$, ^{t} $p = 0.067$. Statistical testing against classification chance level was performed separately in each participant group using one-tailed, one-sample t-test. The results were corrected for multiple comparisons across the word categories, within each group (Bonferroni correction for 3 tests). Error bars represent the standard error of the mean calculated across the results for individual participants in each group. The chance classification level (accuracy = 50%) is marked with black lines. Source data and exact p-values are provided as a Source Data file.

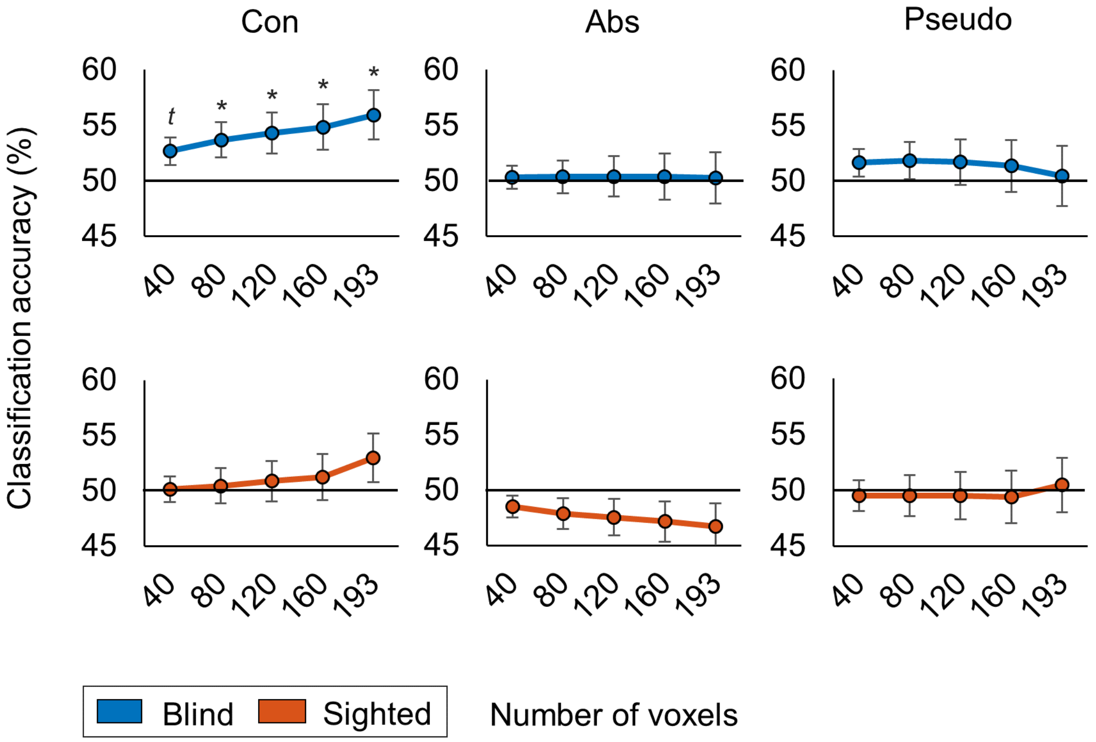

Supplementary Figure 5. Classification of activity patterns for nouns and verbs from specific semantic categories performed in subsets of voxels from the V5/MT mask. Results of support vector machine classification of activity patterns for noun blocks and verb blocks, performed separately for concrete, abstract, and pseudo word categories, in subsets of voxels (40, 80, 120, 160 voxels) that were iteratively drawn from the V5/MT mask in congenitally blind (n = 20) and sighted (n = 20) participants. There were 1000 iterations of the analysis at each level, for each participant, and the results were averaged across the iterations. The classification results obtained in the complete V5/MT mask (193 voxels) are also presented. * $p < 0.05$, $^{t}p = 0.066$. Statistical testing against classification chance level was performed separately in each participant group using one-tailed, one-sample t-test. The results were corrected for multiple comparisons across the word categories, within each group (Bonferroni correction for 3 tests). Error bars represent the standard error of the mean calculated across the results for individual participants in each group. The chance classification level (accuracy = 50%) is marked with black lines. Source data and exact p-values are provided as a Source Data file.

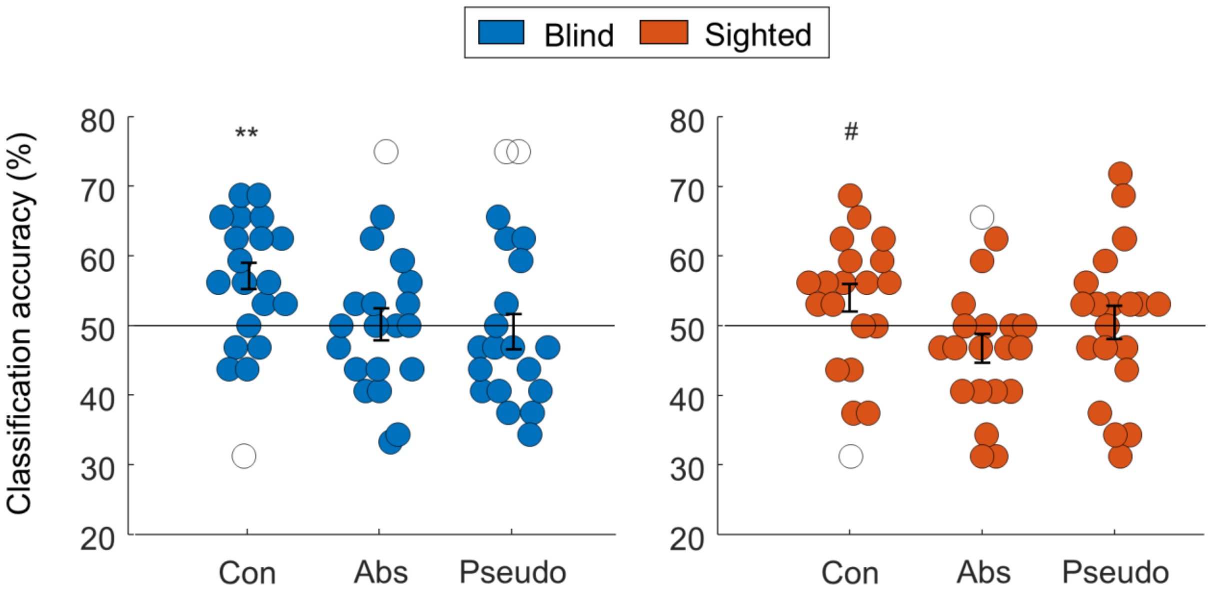

Supplementary Figure 6. Classification of activity patterns for nouns and verbs from specific semantic categories in area V5/MT: individual results. Individual results of support vector machine classification of activity patterns for noun blocks and verb blocks performed separately for concrete, abstract, and pseudo word categories in area V5/MT in congenitally blind (n = 20) and sighted (n = 20) participants. Empty dots mark outliers, defined as values that diverged from the group mean for specific category by more than 2 standard deviations. Tests against classification chance level, results of which are presented in this figure, were performed without these outliers. ** $p < 0.01$. Statistical testing against classification chance level in each participant group was performed using the permutation procedure, in which the actual results were compared with the null distribution of 1000 classification values obtained with the labels of noun blocks and verb blocks randomly reassigned. The results were corrected for multiple comparisons across the word categories, within each group (Bonferroni correction for 3 tests). The effects significant at the uncorrected level are also marked, ($^{\#}$ uncorrected $p = 0.037$). Error bars represent the standard error of the mean calculated across the results for individual participants in each group. The a priori chance classification level (accuracy = 50%) is marked with black lines. Source data and exact p-values are provided as a Source Data file.

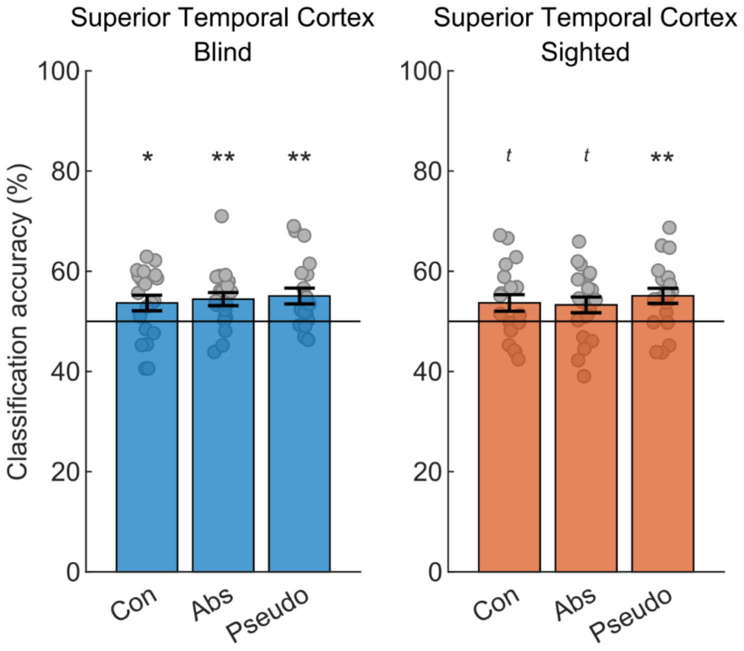

Supplementary Figure 7. Classification of activity patterns for nouns and verbs from specific semantic categories in the superior temporal cortex – analysis in masks with equated number of voxels. Results of support vector machine classification of activity patterns for noun blocks and verb blocks, performed separately for concrete, abstract, and pseudo word categories, in the superior temporal cortex in congenitally blind (n = 20) and sighted (n = 20) participants. The subsets of 193 voxels (which is the size of the V5/MT mask) were randomly drawn from this region's mask (which originally contained 991 voxels). The classification was then performed in these subsets. There were 1000 iterations of this analysis, for each participant, and the results were averaged across the iterations. * $p < 0.05$, ** $p < 0.01$, ^{t} $p < 0.08$. Statistical testing against classification chance level was performed separately in each participant group using one-tailed, one-sample t-test. The results were corrected for multiple comparisons across the word categories, within each group (Bonferroni correction for 3 tests). Error bars represent the standard error of the mean calculated across the results for individual participants in each group. The chance classification level (accuracy = 50%) is marked with black lines. Source data and exact p-values are provided as a Source Data file.

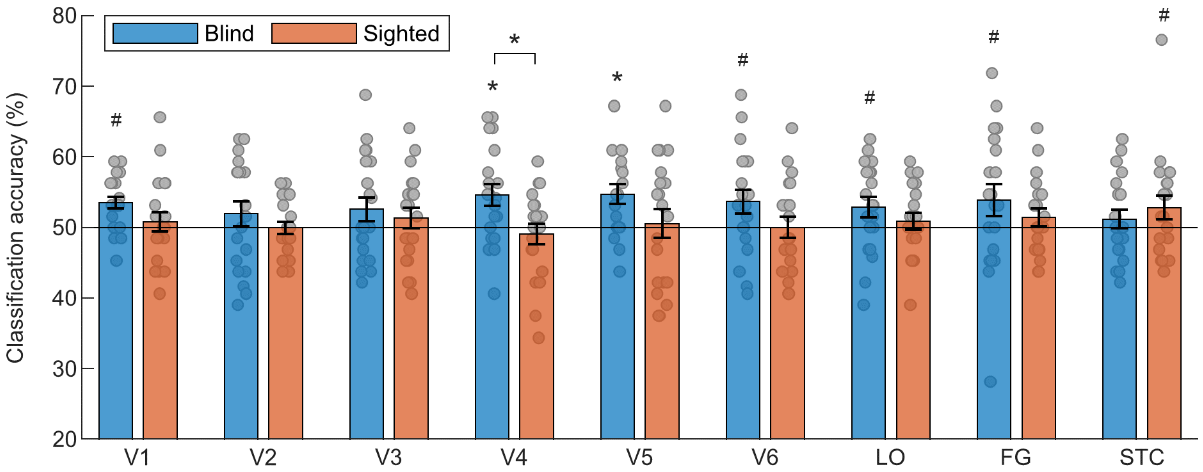

Supplementary Figure 8. Classification of activity patterns for all concrete and all abstract words. Results of support vector machine classification of activity patterns for concrete word blocks and abstract word blocks in the visual areas and the superior temporal cortex in congenitally blind (n = 20) and sighted (n = 20) participants. LO - the lateral occipital area; FG - the fusiform gyrus; STC – the superior temporal cortex. * $p < 0.05$. Statistical testing against classification chance level was performed separately in each participant group using the permutation procedure, in which the actual results were compared with the null distribution of 1000 classification values obtained with the labels of concrete word blocks and abstract word blocks randomly reassigned. The results were corrected for multiple comparisons across the areas, within each group (Bonferroni correction for 9 tests). The between-group tests were performed only in areas V4 and V5, in which significant results were observed in the blind group, using two-tailed, two-sample t-test (Bonferroni correction for 2 tests). The effects significant at the uncorrected level are also marked ($^{\#}$ uncorrected $p < 0.05$). Error bars represent the standard error of the mean calculated across the results for individual participants in each group. The a priori chance classification level (accuracy = 50%) is marked with the black line. Source data and exact p-values are provided as a Source Data file.

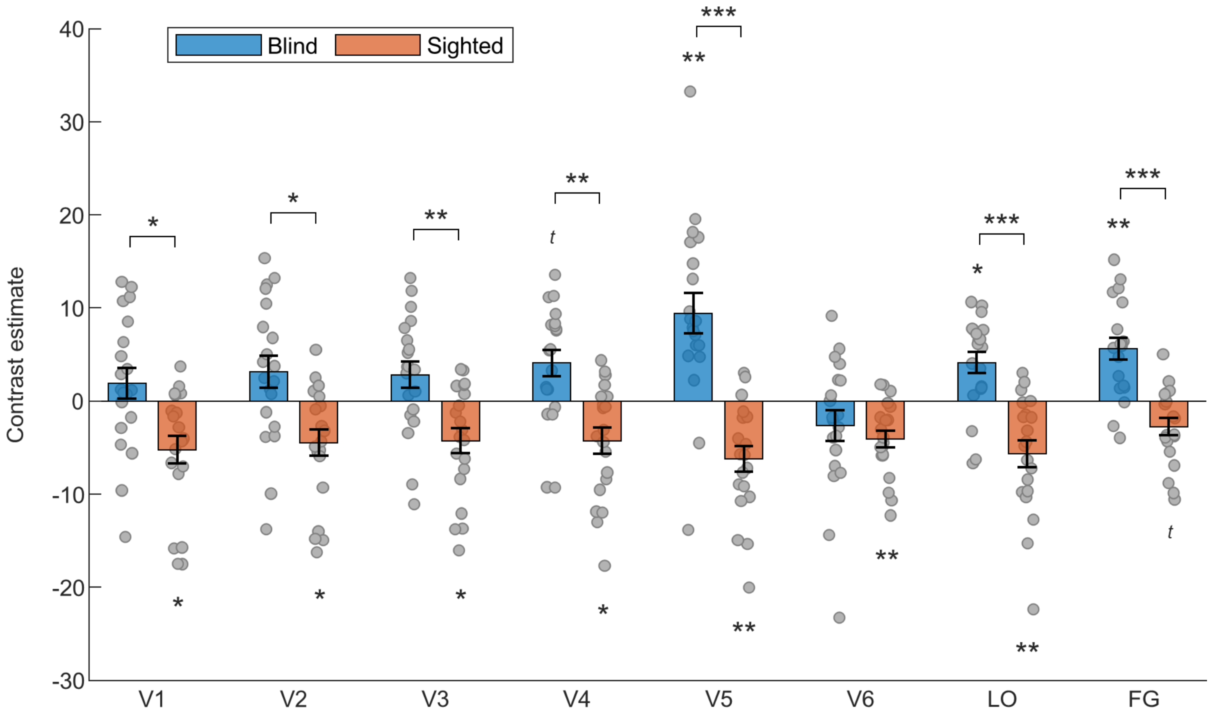

Supplementary Figure 9. Spoken words activated the visual cortex in congenitally blind individuals but deactivated this region in sighted individuals. Average responses to all spoken words and pseudowords, compared to activations during rest periods, in congenitally blind (n = 20) and sighted (n = 20) participants. LO - the lateral occipital area; FG - the fusiform gyrus. * $p < 0.05$, ** $p < 0.01$, *** $p < 0.001$, $^{t}p < 0.08$. Statistical testing against rest-level activations was performed separately in each participant group using two-tailed, one-sample t-test. The results were corrected for multiple comparisons across the visual areas, within each group (Bonferroni correction for 8 tests). The between-group tests were performed in areas in which significant results were observed in either group using two-tailed, two-sample t-test (Bonferroni correction for 8 tests). Error bars represent the standard error of the mean calculated across the results for individual participants in each group. Source data and exact p-values are provided as a Source Data file.

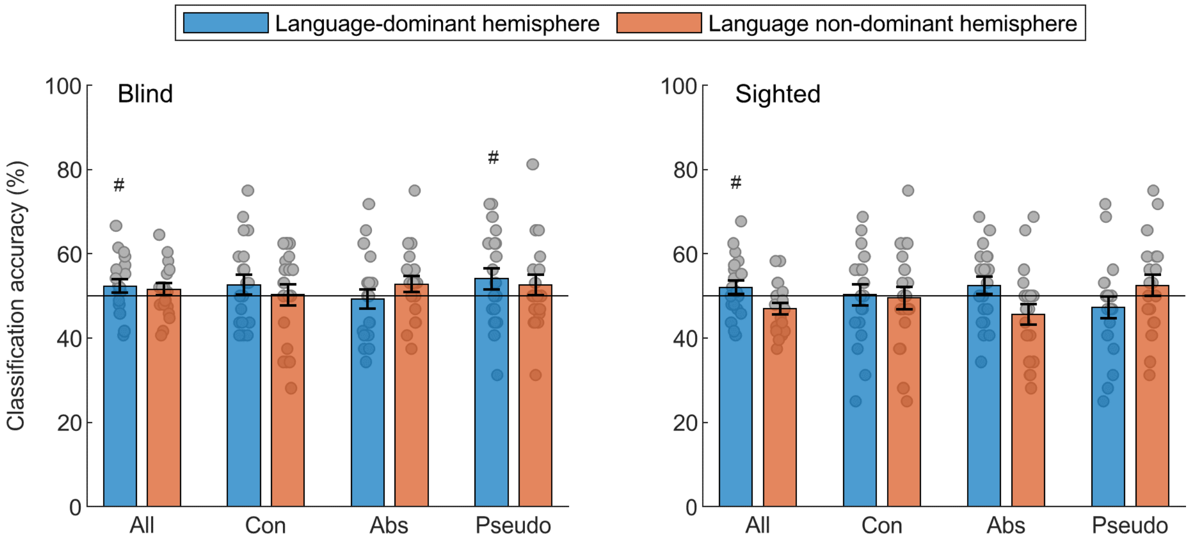

Supplementary Figure 10. Classification of activity patterns for nouns and verbs in area V5/MT: results for the language-dominant and the language non-dominant hemisphere. Results of support vector machine classification of activity patterns for noun blocks and verb blocks in area V5/MT in the language-dominant and the language-nondominant hemisphere in congenitally blind (n = 20) and sighted (n = 20) participants. Classification was performed for all nouns and verbs as well as for nouns and verbs from specific semantic categories. Statistical testing against classification chance level in each participant group was performed using the permutation procedure, in which the actual results were compared with the null distribution of 1000 classification values obtained with the labels of noun blocks and verb blocks randomly reassigned. There were no results that survived the correction for multiple comparisons across the conditions, within each group and hemisphere (Bonferroni correction for 4 tests). # uncorrected $p < 0.05$. Error bars represent the standard error of the mean calculated across the results for individual participants in each group. The a priori chance classification level (accuracy = 50%) is marked with the black line. Source data and exact p-values are provided as a Source Data file.

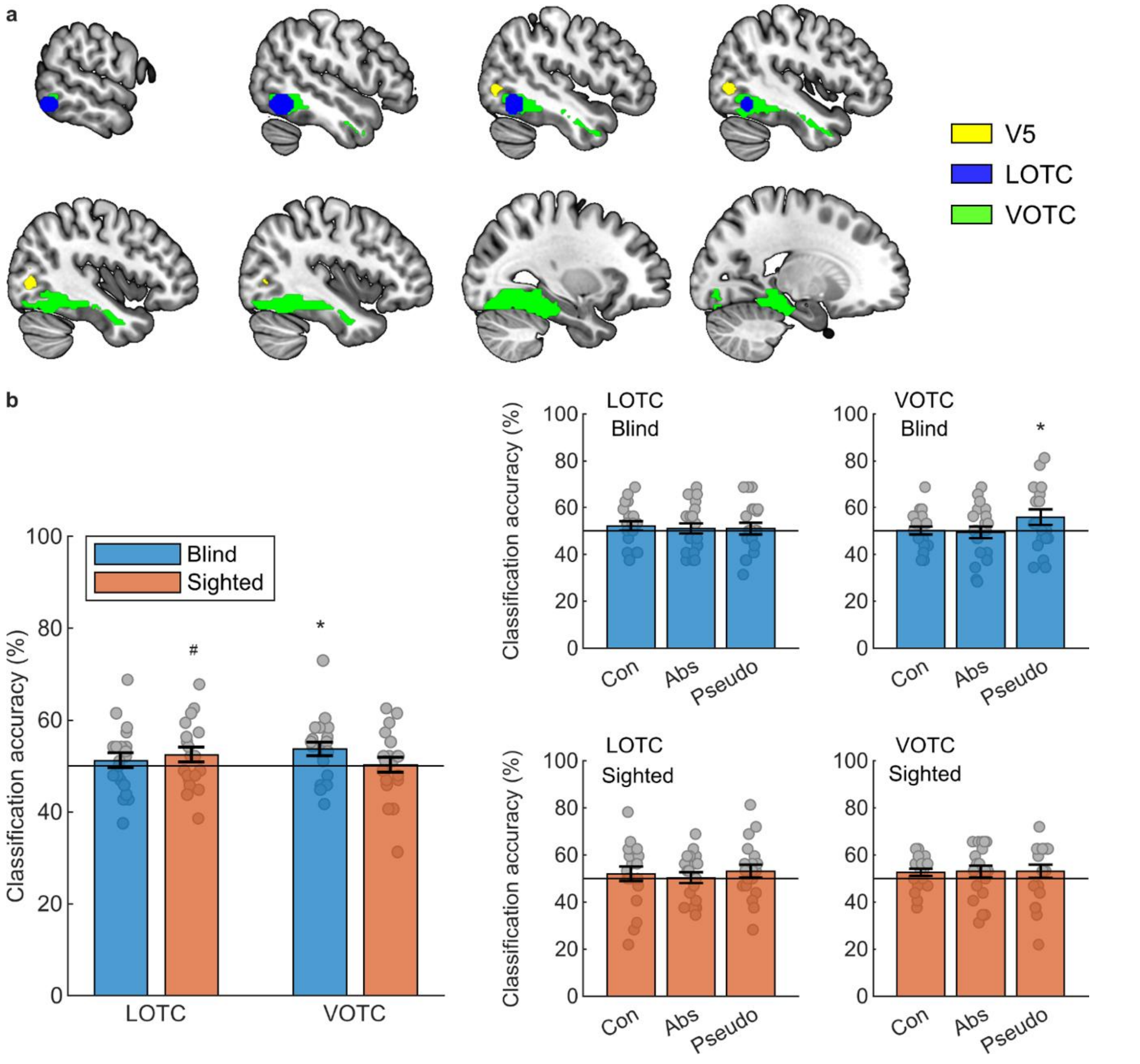

Supplementary Figure 11. Classification of activity patterns for nouns and verbs in the lateral occipitotemporal cortex (LOTC) and the ventral occipitotemporal cortex (VOTC). (A) Masks of LOTC and VOTC, and their location relative to the mask of area V5/MT. The masks were visualized using MRIcroGL (https://www.nitrc.org/projects/mricrogl). (B) Results of support vector machine classification of activity patterns for noun blocks and verb blocks in LOTC and VOTC in congenitally blind (n = 20) and sighted (n = 20) participants. Classification was performed for all nouns and verbs as well as for nouns and verbs from specific semantic categories. Statistical testing against classification chance level was performed separately in each participant group using the permutation procedure, in which the actual results were compared with the null distribution of 1000 classification values obtained with the labels of noun blocks and verb blocks randomly reassigned. The results were corrected for multiple comparisons across the conditions, in each region and group (Bonferroni correction for 4 tests). * $p < 0.05$. The effects significant at the uncorrected level are also marked ($^{\#}$ $p = 0.035$). Error bars represent the standard error of the mean calculated across the results for individual participants in each group. The a priori chance classification level (accuracy = 50%) is marked with black lines. Source data and exact p-values are provided as a Source Data file.

Supplementary Tables

Supplementary Table 1. The linguistic properties of words and pseudo words used in the fMRI experiment. The results are presented at the word category level – averages (and SDs) are presented. The word frequencies are quantified in a form of Zipf scores. Source data are provided as a Source Data file.

Word linguistic properties	Abstract nouns	Abstract verbs	Concrete nouns	Concrete verbs	Pseudo nouns	Pseudo verbs
Frequency – presented word (Zipf)	4,115 (0,307)	3,925 (0,704)	4,091 (0,459)	4,033 (0,572)	-	-
Frequency – target word (Zipf)	3,506 (0,604)	3,46 (0,616)	3,622 (0,664)	3,512 (0,59)	-	-
Syllables – presented word	2,25 (0,608)	2,29 (0,464)	2,25 (0,676)	2,17 (0,381)	2,29 (0,464)	2,25 (0,442)
Syllables – target word	2,75 (0,794)	2,79 (0,588)	2,83 (0,702)	2,71 (0,69)	2,79 (0,658)	2,75 (0,608)

Supplementary Table 2. The results of the behavioral experiment in which sighted participants (n = 15) performed morphological transformations of words and pseudo words used in the fMRI experiments. The task was the same as in the fMRI experiment, but the participants were asked to produce overt responses, which were recorded and analyzed. Response times (from the onset of word presentation to the onset of response) are in milliseconds. Standard deviations (SDs) are reported in parentheses. Source data are provided as a Source Data file.

Word transformations – mean reaction times (SD)	Abstract	Concrete	Pseudo
Nouns	1276 (86)	1251 (67)	1402 (77)
Verbs	1253 (116)	1233 (125)	1389 (161)

Supplementary Table 3. The ratings for words used in the fMRI experiment provided by a group of congenitally blind participants (n = 15). The participants were asked to rate words on three 1 to 7 scales. The results are presented at the word category level – averages (and SDs) are presented.

Word ratings – blind participants	Concreteness	Imageability	Movement connotations
Abstract nouns	3,17 (0,61)	3,43 (0,77)	2,33 (0,68)
Abstract verbs	3,27 (0,73)	3,78 (0,56)	3,65 (1)
Concrete nouns	6,82 (0,23)	6,73 (0,37)	1,58 (0,83)
Concrete verbs	6,28 (0,47)	6,44 (0,32)	6,02 (1,21)

Supplementary Table 4. The ratings for words used in the fMRI experiment provided by a group of sighted participants (n = 46). The participants were asked to rate words on three 1 to 7 scales. The results are presented at the word category level – averages (and SDs) are presented.

Word ratings - sighted participants	Concreteness	Imageability	Movement connotations
Abstract nouns	3,26<br>(0,57)	3,34<br>(0,5)	2,43<br>(0,5)
Abstract verbs	3,49<br>(0,44)	3,57<br>(0,5)	3,44<br>(0,75)
Concrete nouns	6,72<br>(0,21)	6,78<br>(0,17)	1,71<br>(0,59)
Concrete verbs	5,44<br>(0,29)	5,77<br>(0,35)	5,23<br>(1,13)

Supplementary Table 5. Number of voxels included in each visual ROI. LO - the lateral occipital area; FG - the fusiform gyrus.

V1	V2	V3	V4	V5	V6	LO	FG
2441	2031	2680	2070	193	262	2366	2498

Supplementary Table 6. The words and pseudowords used in the fMRI experiment. The presented and the target form in the fMRI experiment are provided. Approximate English translations are also provided for each word.

Word category	Word uninflected form	English translation	Word presented form in the fMRI experiment	Word target form in the fMRI experiment
Abstract nouns	-	decision	decyzja	decyzji
Abstract nouns	-	issue	kłopot	kłopotów
Abstract nouns	-	harm	krzywda	krzywd
Abstract nouns	-	motive	motyw	motywów
Abstract nouns	-	possibility	możliwość	możliwości
Abstract nouns	-	order	nakaz	nakazów
Abstract nouns	-	promise	obietnica	obietnic
Abstract nouns	-	opinion	opinia	opinii
Abstract nouns	-	confidence	pewność	pewności
Abstract nouns	-	trick	podstęp	podstępów
Abstract nouns	-	try	próba	prób
Abstract nouns	-	project	projekt	projektów
Abstract nouns	-	outcome	rezultat	rezultatów
Abstract nouns	-	difference	różnica	różnic
Abstract nouns	-	diagram	schemat	schematów
Abstract nouns	-	secret	sekret	sekretów

Abstract nouns	-	weakness	słabość	słabości
Abstract nouns	-	loose	strata	strat
Abstract nouns	-	style	styl	stylów
Abstract nouns	-	talent	talent	talentów
Abstract nouns	-	deadline	termin	terminów
Abstract nouns	-	property	własność	własności
Abstract nouns	-	ability	zdolność	zdolności
Abstract nouns	-	habit	zwyczaj	zwyczajów
Abstract verbs	bronić	to defend	bronisz	bronimy
Abstract verbs	istnieć	to exist	istnieję	istnieją
Abstract verbs	kończyć	to finish	kończę	kończą
Abstract verbs	należeć	to belong	należę	należą
Abstract verbs	nazywać	to name	nazywasz	nazywamy
Abstract verbs	pamiętać	to remember	pamiętasz	pamiętamy
Abstract verbs	próbować	to try	próbuję	próbują
Abstract verbs	sądzić	to assume	sądzisz	sądzimy
Abstract verbs	służyć	to serve	służę	służą
Abstract verbs	spędzić	to spend	spędzisz	spędzimy
Abstract verbs	spełniać	to fulfill	spełniasz	spełniamy

Abstract verbs	sprawić	to cause	sprawię	sprawią
Abstract verbs	stracić	to lose	stracę	stracą
Abstract verbs	tracić	to lose	tracisz	tracimy
Abstract verbs	trafić	to find a way	trafiasz	trafimy
Abstract verbs	uczynić	to make something happen	uczynię	uczynią
Abstract verbs	udawać	to pretend	udaję	udają
Abstract verbs	umieć	to be able to	umiesz	umiemy
Abstract verbs	wątpić	to doubt	wątpię	wątpią
Abstract verbs	wierzyć	to believe	wierzę	wierzą
Abstract verbs	winić	to blame	winisz	winimy
Abstract verbs	wzywać	to call for	wzywasz	wzywamy
Abstract verbs	zacząć	to begin	zacznę	zaczną
Abstract verbs	zmusić	to force someone to something	zmusisz	zmusimy
Concrete nouns	-	battery	bateria	baterii
Concrete nouns	-	bottle	butelka	butelek
Concrete nouns	-	hand	dłoń	dłoni
Concrete nouns	-	pen	długopis	długopisów

Concrete nouns	-	diploma	dyplom	dyplomów
Concrete nouns	-	carpet	dywan	dywanów
Concrete nouns	-	newspaper	gazeta	gazet
Concrete nouns	-	button	guzik	guzików
Concrete nouns	-	cable	kabel	kabli
Concrete nouns	-	rock	kamień	kamieni
Concrete nouns	-	women	kobieta	kobiet
Concrete nouns	-	computer	komputer	komputerów
Concrete nouns	-	book	książka	książek
Concrete nouns	-	kitchen	kuchnia	kuchni
Concrete nouns	-	chicken	kurczak	kurczaków
Concrete nouns	-	flower	kwiat	kwiatów
Concrete nouns	-	writing	napis	napisów
Concrete nouns	-	container	pojemnik	pojemników

Concrete nouns	-	towel	ręcznik	ręczników
Concrete nouns	-	table	stolik	stolików
Concrete nouns	-	dress	sukienka	sukienek
Concrete nouns	-	plate	talerz	talerzy
Concrete nouns	-	telephone	telefon	telefonów
Concrete nouns	-	face	twarz	twarzy
Concrete verbs	biegać	to run	biegasz	biegamy
Concrete verbs	chodzić	to walk	chodzisz	chodzimy
Concrete verbs	ćwiczyć	to exercise	ćwiczę	ćwiczą
Concrete verbs	dotknąć	to touch	dotkniesz	dotkniemy
Concrete verbs	gadać	to talk	gadasz	gadamy
Concrete verbs	jechać	to drive	jadę	jadą
Concrete verbs	kopać	to kick	kopię	kopią
Concrete verbs	krzyczeć	to scream	krzyczysz	krzyczymy
Concrete verbs	łapać	to catch	łapię	łapią
Concrete verbs	mówić	to talk	mówię	mówią
Concrete verbs	otworzyć	to open	otworzę	otworzą

Concrete verbs	pisać	to write	piszę	piszą
Concrete verbs	pójść	to go	pójdę	pójdą
Concrete verbs	przejść	to go across	przejdę	przejdą
Concrete verbs	rzucać	to throw	rzucasz	rzucamy
Concrete verbs	siedzieć	to sit	siedzisz	siedzimy
Concrete verbs	skakać	to jump	skaczę	skaczą
Concrete verbs	słyszeć	to hear	słyszysz	słyszymy
Concrete verbs	śpiewać	to sing	śpiewasz	śpiewamy
Concrete verbs	tańczyć	to dance	tańczę	tańczą
Concrete verbs	uciekać	to run away	uciekasz	uciekamy
Concrete verbs	usiąść	to sit	usiądziesz	usiądziemy
Concrete verbs	wstawać	to get up	wstaję	wstają
Concrete verbs	wychodzić	to go out	wychodzisz	wychodzimy
Pseudo nouns	-	-	buzeta	buzet
Pseudo nouns	-	-	czowier	czowierów
Pseudo nouns	-	-	czukoć	czukoci
Pseudo nouns	-	-	dejaźń	dejaźni
Pseudo nouns	-	-	doraść	doraści
Pseudo nouns	-	-	gaboraz	gaborazów

Pseudo nouns	-	-	gauwa	guaw
Pseudo nouns	-	-	katela	katel
Pseudo nouns	-	-	koczak	koczaków
Pseudo nouns	-	-	komyba	komyb
Pseudo nouns	-	-	memont	memontów
Pseudo nouns	-	-	minotyw	minotywów
Pseudo nouns	-	-	mizak	mizaków
Pseudo nouns	-	-	mokat	mokatów
Pseudo nouns	-	-	moraba	morab
Pseudo nouns	-	-	pazach	pazachów
Pseudo nouns	-	-	powań	powani
Pseudo nouns	-	-	rabak	rabaków
Pseudo nouns	--	-	rekretyl	rekretylów
Pseudo nouns	-	-	unaka	unak
Pseudo nouns	-	-	wajaść	wajaści
Pseudo nouns	-	-	wiepoć	wiepoci
Pseudo nouns	-	-	wrópot	wrópotów
Pseudo nouns	-	-	zatyl	zatylów
Pseudo verbs	brożyć	-	brożę	brożą

Pseudo verbs	choczyć	-	choczę	choczą
Pseudo verbs	ćwiszyć	-	ćwiszę	ćwiszą
Pseudo verbs	czyszyć	-	czyszę	czyszą
Pseudo verbs	dazić	-	dazisz	dazimy
Pseudo verbs	doporzyć	-	doporzę	doporzą
Pseudo verbs	jamaszyć	-	jamaszysz	jamaszymy
Pseudo verbs	kaskać	-	kaskasz	kaskaszamy
Pseudo verbs	kódzić	-	kódzę	kódzą
Pseudo verbs	ławić	-	ławisz	ławimy
Pseudo verbs	pakoczyć	-	pakoczę	pakoczą
Pseudo verbs	piświć	-	piswię	piswią
Pseudo verbs	słunić	-	słunisz	słunimy
Pseudo verbs	spężyć	-	spężę	spężą
Pseudo verbs	śpierzyć	-	śpierzysz	spierzymy
Pseudo verbs	spamać	-	spramasz	spramamy
Pseudo verbs	szesłać	-	szesłasz	szesłamy
Pseudo verbs	toić	-	toisz	toimy
Pseudo verbs	unajać	-	unaję	unają
Pseudo verbs	wadać	-	wadasz	wadamy

Pseudo verbs	wazerać	-	wazerasz	wazeramy
Pseudo verbs	wojić	-	woję	wojią
Pseudo verbs	żelać	-	żelasz	żelamy
Pseudo verbs	złobujeć	-	złobuję	złobują
