# Supplementary material for: Neural representation of nouns and verbs in congenitally blind and sighted individuals
Source: Nat Commun. 2025 Aug 29;16:8090. doi: 10.1038/s41467-025-63423-0 (PMC12397302; doi:10.1038/s41467-025-63423-0)
Supplement: Supplementary file 2 — Reporting Summary [file 41467_2025_63423_MOESM2_ESM.pdf]

nature portfolio

Corresponding author(s): Lukasz Bola
Last updated by author(s): Jul 22, 2025

# Reporting Summary

Nature Portfolio wishes to improve the reproducibility of the work that we publish. This form provides structure for consistency and transparency in reporting. For further information on Nature Portfolio policies, see our Editorial Policies and the Editorial Policy Checklist.

## Statistics

For all statistical analyses, confirm that the following items are present in the figure legend, table legend, main text, or Methods section.

| n/a | Confirmed | |
|---|---|---|
| ☐ | ☒ | The exact sample size (*n*) for each experimental group/condition, given as a discrete number and unit of measurement |
| ☐ | ☒ | A statement on whether measurements were taken from distinct samples or whether the same sample was measured repeatedly |
| ☐ | ☒ | The statistical test(s) used AND whether they are one- or two-sided<br>*Only common tests should be described solely by name; describe more complex techniques in the Methods section.* |
| ☐ | ☒ | A description of all covariates tested |
| ☐ | ☒ | A description of any assumptions or corrections, such as tests of normality and adjustment for multiple comparisons |
| ☐ | ☒ | A full description of the statistical parameters including central tendency (e.g. means) or other basic estimates (e.g. regression coefficient) AND variation (e.g. standard deviation) or associated estimates of uncertainty (e.g. confidence intervals) |
| ☐ | ☒ | For null hypothesis testing, the test statistic (e.g. *F*, *t*, *r*) with confidence intervals, effect sizes, degrees of freedom and *P* value noted<br>*Give P values as exact values whenever suitable.* |
| ☒ | ☐ | For Bayesian analysis, information on the choice of priors and Markov chain Monte Carlo settings |
| ☒ | ☐ | For hierarchical and complex designs, identification of the appropriate level for tests and full reporting of outcomes |
| ☐ | ☒ | Estimates of effect sizes (e.g. Cohen's *d*, Pearson's *r*), indicating how they were calculated |

*Our web collection on statistics for biologists contains articles on many of the points above.*

## Software and code

Policy information about availability of computer code

| | |
|---|---|
| Data collection | PsychoPy 3.0.12b: The presentation of stimuli (the fMRI study and the behavioral studies) |
| Data analysis | Dcm2niix (v1.0.20220720): Conversion of neuroimaging data from the DICOM format to the NIFTI format.<br>SPM 12 (v. 6906), CONN 21b. Matlab 2022a: Neuroimaging data preprocessing<br>CosmoMVPA (v.1.1.0), LIBSVM (v. 3.23), SPM 12, Matlab 2022a, SPSS 25: Multi-voxel pattern classification analyses<br>SPM 12 (v. 6906), Matlab 2022a, SPSS 25: The univariate analyses<br>JuBrain Anatomy Toolbox (v. 3.0): ROI definitions<br>Chronset, SPSS 25: Behavioral data analysis<br>DataViz Matlab toolbox (v. 3.2.4), BrainNet Viewer (v. 1.7), MRIcroGL (v. 1.2.20220620): Data visualization |

For manuscripts utilizing custom algorithms or software that are central to the research but not yet described in published literature, software must be made available to editors and reviewers. We strongly encourage code deposition in a community repository (e.g. GitHub). See the Nature Portfolio guidelines for submitting code & software for further information.

## Data

Policy information about availability of data

All manuscripts must include a data availability statement. This statement should provide the following information, where applicable:

- Accession codes, unique identifiers, or web links for publicly available datasets
- A description of any restrictions on data availability
- For clinical datasets or third party data, please ensure that the statement adheres to our policy

We provide the following data availability statement in the manuscript:
The neuroimaging data generated in this study have been deposited at the Open Science Framework (https://osf.io/vqwuk/). Source data are provided with this paper.

## Research involving human participants, their data, or biological material

Policy information about studies with human participants or human data. See also policy information about sex, gender (identity/presentation), and sexual orientation and race, ethnicity and racism.

| | |
|---|---|
| Reporting on sex and gender | We recruited the human participants of various sexes. The sex of participants was determined based on self-report. As reported in the paper, the blind group (9 males, 11 females) and the sighted group (6 males, 14 females) were matched for sex. The sex-based analyses were not performed because of relatively low sample size and lack of a priori hypotheses concerning the impact of this dimension on the results. The individual data marked for participants' sex, age, handedness, and education level are provided in the source data file. |
| Reporting on race, ethnicity, or other socially relevant groupings | We did not recruit or group participants based on their race, ethnicity, or other socially relevant dimensions. |
| Population characteristics | We recruited 20 congenitally blind subjects (9 males, 11 females, mean age = 35.65 y, SD = 7.81 y, average length of education = 14.8 y, SD = 2.35 y) and 20 sighted subjects (6 males, 14 females, mean age = 35 y, SD = 8.58 y, average length of education = 15.4 y, SD = 2.04 y). All except two participants were right-handed, and the remaining two participants (one blind, one sighted) were left-handed. The blind and the sighted groups were matched for age, sex, handedness, and years of education (Mann-Whitney and Chi-square tests, all p values > 0.25). In the blind group, blindness had a variety of causes, including retinopathy of prematurity, glaucoma, Leber's congenital amaurosis, optic nerve hypoplasia, or unknown causes. Most blind participants reported to have some light perception, but no object or contour vision. One blind participant reported to have some form of contour vision, which, however, was not precise enough to be functional. All subjects in both groups were native Polish speakers, had normal hearing, and had no history of neurological disorders. All subjects had no contraindications to the MRI, gave written informed consent and were paid for participation. |
| Recruitment | We first recruited the congenitally blind participants. The recruitment was conducted in social medias and using the network of blind participants developed for the previous studies. We were enrolling all congenitally blind participants who volunteered and met our recruitment criteria described above, until we achieved our target sample size (n=20). We then started recruiting the sighted participants. The recruitment was conducted in social medias. The sighted participants were chosen so that the sighted group matched the blind group in demographic dimensions described above. |
| Ethics oversight | The study was approved by the ethics committee of Institute of Psychology, Polish Academy of Sciences. |

Note that full information on the approval of the study protocol must also be provided in the manuscript.

# Field-specific reporting

Please select the one below that is the best fit for your research. If you are not sure, read the appropriate sections before making your selection.

☒ Life sciences ☐ Behavioural & social sciences ☐ Ecological, evolutionary & environmental sciences

For a reference copy of the document with all sections, see nature.com/documents/nr-reporting-summary-flat.pdf

# Life sciences study design

All studies must disclose on these points even when the disclosure is negative.

| | |
|---|---|
| Sample size | Target sample size was a prori set to n = 20 per group. No statistical calculations were performed - we worked with a unique population (congenitally blind individuals) and we reasoned that n = 20 is the maximal sample size we were likely to achieve. Notably, in virtually all published studies of language responses in the visual cortex of blind individuals, the sample sizes were comparable to or smaller than in our study. Thus, we were confident that our target sample size is sufficient to produce meaningful effects. |
| Data exclusions | In one blind participant, the data collection during the final, fourth fMRI run was interrupted by an alarm and ensuing evacuation of the research facility. This run was excluded from the analysis. Apart from that, no data were excluded. |

| | |
|---|---|
| Replication | No replication of our fMRI study was attempted. We did, however, perform a number of control analyses and conservatively corrected our data for multiple comparisons to ensure that the effects we report are robust. |
| Randomization | We a priori decided to recruit two groups - the sighted participants and the congenitally blind participants. In line with best practices in our field, the two groups were matched for a number of factors that could potentially influence the results, such as age, sex, handedness, or education.<br>We studied volunteers, which raises the possibility of self-selection bias. However, we consider such bias to be unlikely to affect relatively basic biological processes (activations in the visual areas) that we investigate in our study. |
| Blinding | The investigators were not blinded. During the data collections, this was impossible - we studied two visibly different groups, the sighted and the congenitally blind participants. We also did not use blinding during the data analysis, mostly for practical reasons. However, we followed exactly the same analytical protocol during the analysis of data from both groups. |

# Reporting for specific materials, systems and methods

We require information from authors about some types of materials, experimental systems and methods used in many studies. Here, indicate whether each material, system or method listed is relevant to your study. If you are not sure if a list item applies to your research, read the appropriate section before selecting a response.

## Materials & experimental systems

| n/a | Involved in the study | |
|---|---|---|
| ☒ | ☐ | Antibodies |
| ☒ | ☐ | Eukaryotic cell lines |
| ☒ | ☐ | Palaeontology and archaeology |
| ☒ | ☐ | Animals and other organisms |
| ☒ | ☐ | Clinical data |
| ☒ | ☐ | Dual use research of concern |
| ☒ | ☐ | Plants |

## Methods

| n/a | Involved in the study | |
|---|---|---|
| ☒ | ☐ | ChIP-seq |
| ☒ | ☐ | Flow cytometry |
| ☐ | ☒ | MRI-based neuroimaging |

# Plants

| | |
|---|---|
| Seed stocks | *Report on the source of all seed stocks or other plant material used. If applicable, state the seed stock centre and catalogue number. If plant specimens were collected from the field, describe the collection location, date and sampling procedures.* |
| Novel plant genotypes | *Describe the methods by which all novel plant genotypes were produced. This includes those generated by transgenic approaches, gene editing, chemical/radiation-based mutagenesis and hybridization. For transgenic lines, describe the transformation method, the number of independent lines analyzed and the generation upon which experiments were performed. For gene-edited lines, describe the editor used, the endogenous sequence targeted for editing, the targeting guide RNA sequence (if applicable) and how the editor was applied.* |
| Authentication | *Describe any authentication procedures for each seed stock used or novel genotype generated. Describe any experiments used to assess the effect of a mutation and, where applicable, how potential secondary effects (e.g. second site T-DNA insertions, mosiacism, off-target gene editing) were examined.* |

# Magnetic resonance imaging

## Experimental design

| | |
|---|---|
| Design type | We used a task block design |
| Design specifications | In the fMRI, we presented participants with blocks of 6 words belonging to the same category. There were 6 word categories: abstract nouns, abstract verbs, concrete nouns, concrete verbs, pseudo nouns, and pseudo verbs. For each word category, there were 4 blocks per run and 16 blocks in total, in the whole study. Each block lasted 15 s. Different words were used in each fMRI run. |
| Behavioral performance measures | During the fMRI study, the subjects were asked to transform the words from singular to plural forms. They were asked to perform the task mentally, i.e., without an overt response. We did not ask for an overt responses because, first, recordings of spoken responses would be difficult in the noisy MRI environment, and second, overt responses would likely cause significant head motions and, consequently, interfere with data collection. During the fMRI study, we ensured that the participants were awake, attentive, and able to perform the task, after each fMRI run. Furthermore, we ensured that our task worked as expected during a separate behavioral study, in which the overt responses were required and recorded. |

## Acquisition

| | |
|---|---|
| Imaging type(s) | functional and structural |
| Field strength | 3T |
| Sequence & imaging parameters | Data were acquired on a 3-T Siemens Trio Tim MRI scanner using a 32-channel head coil at the Laboratory of Brain Imaging in Nencki Institute of Experimental Biology in Warsaw. Functional data were acquired using a multiband sequence with the following parameters: 60 slices, phase encoding direction from posterior to anterior; voxel size: 2,5 mm3; TR = 1.41 s; TE: 30.4 ms; multiband factor: 3. Before the start of the first functional run, T1-weighted anatomic scans were acquired using MPRAGE sequence with the following parameters: 208 slices, phase encoding direction from anterior to posterior; voxel size: 0,8 mm3; TR = 2.5 s; TE: 21.7 ms. |
| Area of acquisition | Data from the whole brain were collected |

Diffusion MRI ☐ Used ☒ Not used

## Preprocessing

| | |
|---|---|
| Preprocessing software | The MRI data were converted from the DICOM format to the NIFTI format using the dcm2niix. Then, the preprocessing was performed using SPM 12 and CONN 21b toolbox, running on MATLAB R2022a. |
| Normalization | We used a direct segmentation and normalization approach, as implemented in SPM 12 and CONN 21b. The functional and anatomical data were segmented into gray matter, white matter, and CSF tissue classes and normalized into standard Montreal Neurological Institute (MNI) space using unified segmentation and normalization procedure |
| Normalization template | Montreal Neurological Institute (MNI) template, as implemented in SPM 12 and CONN 21b |
| Noise and artifact removal | Functional realignment of all functional images, with standard parameters, as implemented in SPM 12 and CONN 21b |
| Volume censoring | No volume censoring was used |

## Statistical modeling & inference

Model type and settings

First level
Two fixed-effect statistical models were created for each subject. For the multi-voxel pattern classification analysis, the data were modeled at the level of single blocks (24 predictors per run, one for each block). Additionally, transformation cues were modeled as conditions of no interest (8 predictors per run, one for each occurrence of the cue). For the univariate analysis, the data were modeled at the level of word categories (6 predictors per run, one for each word category, 1 predictor of no interest per run for cues). Signal time course was modeled using a general linear model by convolving a canonical hemodynamic response function with the time series of predictors. Six movement parameter regressors obtained during the preprocessing were added to the models. An inclusive high-pass filter was used (378 s, approximately 2 cycles per run) to remove drifts from the signal while ensuring that effects specific to each word category were not filtered out from the data. Autocorrelations were accounted for using autoregressive AR(1) model. Individual beta maps, contrast maps, and t-maps were computed for each experimental block/condition, relative to rest periods.

Second level - MVPA
In the multi-voxel pattern analysis, the contrast maps and a support vector machine algorithm were used for classification, which was performed at the level of individual data.
In the ROI analysis, testing the group average classification accuracy against chance level was then performed in the permutation procedure (i.e., comparing the actual results with the null distribution of 1000 results obtained when the labels of experimental conditions were randomly assigned to the fMRI blocks), that is, without a formal statistical model. The analyses of effects in specific subsets of voxels drawn from the anatomical masks (Supplementary Figures 3-5 and Supplementary Fig. 7) were already based on permutations. Thus, in these specific analyses, using the permutation procedure for significance testing was not practical, and testing against classification chance level was performed with one-tailed one-sample t-tests. Comparison of results across conditions, ROIs, and groups was performed with two-sided t tests and with ANOVAs, calculated in SPSS 25.
In the searchlight analysis, the individual classification accuracies were entered into SPM group models. One-tailed one-sample t-tests were used to compare the results of each searchlight analysis with chance level, separately in the blind and the sighted group. One-tailed two-sample t-tests were used to compare the results between groups.

Second level – Univariate analysis
In the whole-brain analysis, the contrast estimates for all experimental conditions, relative to rest periods, were averaged at the single-subject level. Then, the average activation maps were entered into SPM one-tailed one-sample t-tests, performed separately for each group, and into the SPM one-tailed two-sample t-test, which tested for the between-group differences. The whole-brain analysis was followed by the ROI analysis. Two-tailed one-sample t-tests, two-tailed two-sample t-tests, and ANOVAs, calculated in SPSS 25 were used to analyze the data.

Effect(s) tested

In the MVPA:
1) We first classified activity patterns for noun and verb blocks when all word categories (concrete, abstract, pseudo) were included in the analysis.
2) Then, we performed classifications of activity patterns for noun and verb blocks separately for each word category.
3) Additionally, we also classified activity patterns for concrete and abstract word blocks.

In the univariate analysis:
1) We first performed the whole-brain univariate analysis, in which we compared the activation induced by all words and pseudowords to rest periods, in both participant groups.
2) This analysis was followed by more detailed ROI analysis of the same effects (all stimuli vs. rest) in the visual cortex.
3) Finally, we performed the ROI analysis of activations for each experimental condition, relative to rest periods, in the area V5/MT, in which the multi-voxel pattern classification showed significant effects.

Specify type of analysis: ☐ Whole brain ☐ ROI-based ☒ Both

Anatomical location(s): The region of interest (ROI) analyses were performed using maps from the JuBrain Anatomy Toolbox. The analyses in the visual cortex were performed in all occipital and occipitotemporal regions delineated in the Toolbox. To reduce a number of tests, subregions were combined (e.g., areas V3d and V3v were combined in area V3). The analysis in the superior temporal cortex was performed using the area TE3 mask.

Statistic type for inference
(See Eklund et al. 2016)

Statistical threshold used in the ROI analyses: $p < 0.05$, corrected for multiple comparison using Bonferroni correction
Statistical threshold used in the whole-brain and searchlight analyses: parametric voxelwise $p < 0.001$, corrected for multiple comparisons using family-wise error cluster (FWEc) correction approach, as implemented in SPM 12

Correction

Classic Bonferroni correction approach was used in the ROI analyses
Family-wise error cluster (FWEc) correction approach, as implemented in SPM 12, was used in the whole-brain analyses. The voxelwise threshold at which the correction was performed was set to $p < 0.001$, in line with recommendations of Eklund et al.

## Models & analysis

| n/a | Involved in the study |
| --- | --- |
| ☒ | ☐ Functional and/or effective connectivity |
| ☒ | ☐ Graph analysis |
| ☒ | ☐ Multivariate modeling or predictive analysis |
